# Diagnostic heterogeneity in scrub typhus serology: A scoping review of IFA thresholds and regional standardisation needs (2005–2024)

**DOI:** 10.1371/journal.pntd.0013540

**Published:** 2025-10-22

**Authors:** Khanh Kim Le, Kartika Saraswati, Jantana Wongsantichon, Nicholas P. J. Day, Stuart D. Blacksell

**Affiliations:** 1 Mahidol Oxford Tropical Medicine Research Unit, Faculty of Tropical Medicine, Mahidol University, Bangkok, Thailand; 2 Saw Swee Hock School of Public Health, National University of Singapore and National University Health System, Singapore, Singapore; 3 Oxford University Clinical Research Unit Indonesia, Faculty of Medicine, Universitas Indonesia, Jakarta, Indonesia; 4 Centre for Tropical Medicine and Global Health, Nuffield Department of Medicine, Nuffield Department of Medicine Research Building, University of Oxford, Oxford, United Kingdom; Huadong Research Institute for Medicine and Biotechniques, CHINA

## Abstract

**Background:**

The indirect immunofluorescence assay (IFA) remains the most widely used reference method for diagnosing scrub typhus. However, inconsistent cut-off thresholds and strain selections across studies hinder standardisation and complicate cross-regional comparisons. This scoping review examines diagnostic heterogeneity in IFA-based scrub typhus serology and assesses the need for region-specific standardisation.

**Methods:**

We conducted a systematic search of peer-reviewed literature published between January 2005 and May 2024 across PubMed, Scopus, and Web of Science databases. Studies were included if they employed IFA for diagnosing or conducting seroepidemiological investigations of scrub typhus and reported specific IgM or IgG titre thresholds. Data were extracted regarding IFA methodology, antigen strains used, titre cut-offs for positivity, sample populations, and geographic settings. The studies were mapped and synthesised to identify trends, methodological diversity, and regional variation in IFA practices.

**Results:**

A total of 84 studies met the inclusion criteria, covering 16 countries across Asia-Pacific and South Asia. The diagnostic cut-off titres for IgM ranged widely from 1:10 to 1:25,600, with considerable variability both within and between countries. Many studies lacked a clearly stated rationale for threshold selection or did not reference region-specific validation. Antigen panels were often limited to prototype strains (e.g., Karp, Gilliam, Kato), with few incorporating locally circulating genotypes. Seroprevalence estimates were significantly influenced by the selected cut-off and antigen composition. Only a minority of studies employed standardised or validated thresholds aligned with regional disease endemicity.

**Conclusion:**

This review underscores significant heterogeneity in IFA cut-offs and strain selection in scrub typhus serology, highlighting the urgent need for regionally validated diagnostic standards. Greater harmonisation of IFA protocols, including rational cut-off determination and inclusion of locally relevant strains, is crucial for improving diagnostic accuracy and informing surveillance and public health strategies.

## Author summary

Scrub typhus is a serious disease caused by bacteria transmitted through the bites of infected mites. It is common in many parts of Asia and the Pacific. Diagnosing scrub typhus accurately is important for patient care and disease monitoring. Still, the most commonly used test—the indirect immunofluorescence assay (IFA)—varies widely in how it is used across different countries and laboratories. This review looked at nearly 20 years of published research to understand how other studies have used the IFA test. We found that there is no consistent agreement on what level of antibodies should count as a “positive” result, and many studies used different strains of the bacteria that may not reflect what is actually circulating in the region. These differences make it hard to compare results across countries or over time. The findings show a clear need for more consistent, regionally appropriate guidelines on how to perform and interpret this test. Improving standardisation will help ensure more accurate diagnoses, better tracking of disease patterns, and stronger public health responses in areas where scrub typhus is common.

## Introduction

Scrub typhus is a febrile illness primarily caused by the bacterium *Orientia tsutsugamushi*, representing a significant yet often neglected threat to global health [1]. Scrub typhus was historically considered endemic only to the “tsutsugamushi triangle,” which includes parts of South and Southeast Asia, northern Australia, and the western Pacific. However, it is now recognised as an emerging global infectious disease, with reports of *Orientia*-like pathogens from Africa, South America, the Middle East, and Europe underscoring its geographic expansion [2]. This vector-borne zoonosis is transmitted to humans through the bite of infected larval-stage mites (chiggers). Individuals working in rural or agricultural environments are disproportionately affected, with prevalence rates in these populations reaching as high as 48% in endemic regions [3]. Untreated scrub typhus can have mortality rates up to 70%, with a median of 6%, depending on the strain and healthcare capacity [4]. Even with treatment, delays in diagnosis can lead to severe complications like organ failure, straining healthcare systems in resource-limited areas [4].

Accurate and timely diagnosis is critical to reducing morbidity and mortality associated with scrub typhus. However, clinical diagnosis remains challenging due to non-specific symptoms that overlap with those of other febrile illnesses, such as dengue, typhoid, and leptospirosis, making laboratory-based diagnostics essential for confirmation [5]. Real-time PCR remains the most suitable method for diagnosing acute scrub typhus; however, accurate diagnosis relies on the use of the buffy coat or eschar as the optimal sample type, ideally collected during the early rickettsemic phase of infection [6,7]. For serological diagnosis, whether in acute or retrospective cases, the enigmatic indirect immunofluorescence assay (IFA) continues to be regarded as the reference standard for scrub typhus and other rickettsial diseases [8]. The increasingly popular commercial [9,10] or in-house [11,12] ELISA-based tests, which are simpler to perform, potentially offer greater flexibility and throughput; however, they tend to produce results that are less intuitive to interpret, hence the reliance on IFA for reference serology. For the diagnosis of scrub typhus infection, the IFA is ideally performed on paired acute and convalescent samples, relying on a four-fold or greater rise in titre to indicate an active infection [8]. However, it is often impractical to collect paired samples, resulting in a reliance on the IFA titre from a single serum sample for diagnosis. This approach has significant limitations because of geographic variation in scrub typhus endemicity, which results in differing levels of background immunity, influenced by the frequency of pathogen exposure and the longevity of antibody responses [8,13,14]. Furthermore, IFA inter-laboratory variability may arise from differences in laboratory infrastructure and antigen strain composition. These factors could potentially compromise diagnostic accuracy, delay treatment, and limit the comparability of surveillance data across regions. Aside from acute or retrospective diagnosis, seroepidemiology studies also suffer from the absence of a standardised, region-specific cut-off, potentially leading to inaccurate seroprevalence estimates [13].

A 2007 systematic review by Blacksell *and colleagues* [8] highlighted these variations and called for evidence-based local cut-offs informed by epidemiological data, emphasising the reliability of dynamic criteria, such as a four-fold or greater rise in titres between acute and convalescent samples. Nearly two decades after this original study, we have systematically reviewed articles published between 2005 and 2024 to determine the situation. Specifically, this review investigated four key areas: (1) the range and variation of diagnostic cut-off thresholds for scrub typhus IgM and IgG; (2) the methodological quality and transparency of IFA reporting; (3) geographic and epidemiological influences on diagnostic practices; and (4) the composition and reporting of antigenic strains used in IFA assays. Beyond mapping current practices, our findings highlight gaps that directly inform policy and operational priorities—from developing region-specific cut-offs, to implementing standardised antigen panels, to aligning IFA with ELISA platforms. These insights aim to support national and regional efforts to harmonise diagnostic criteria, strengthen surveillance systems, and guide treatment strategies for this neglected tropical disease as it continues to emerge in new regions.

## Methods

This review adheres closely to the framework established by Blacksell *and colleagues* [8] to ensure methodological consistency and comparability with prior research. Key components of the original protocol, including article selection processes, data extraction, and inclusion/exclusion criteria, were preserved. The expanded scope of this updated review includes articles from July 2005 to September 2024, enabling a comprehensive assessment of advances in diagnostic practices since the original review.

### Literature search

A systematic search was conducted in PubMed, Embase, and supplementary databases. The following search terms were applied: ((scrub typhus OR tsutsugamushi) AND (diagnosis OR diagnostic) AND (immunofluorescen* OR fluoresce* OR IFA))

### Study selection

**Fig 1:**
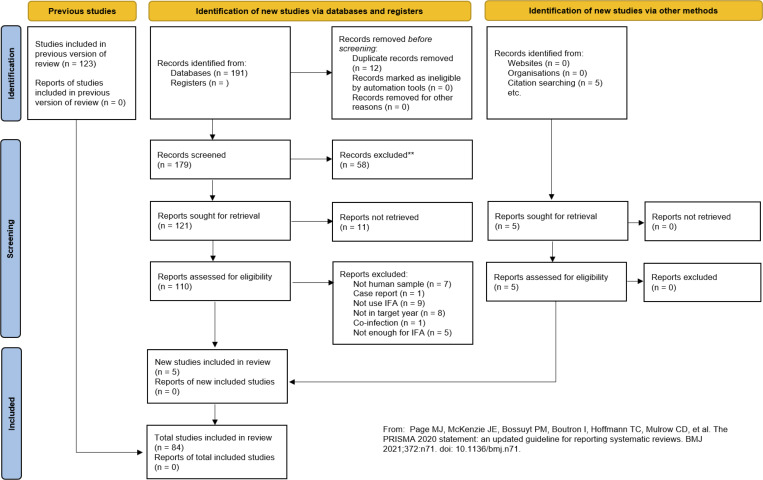
Flowchart of the article selection process.

The Preferred Reporting Items for Systematic Reviews and Meta-Analyses (PRISMA) guidelines were used to ensure a transparent and reproducible study selection process (Fig 1). Eligible articles met the following inclusion criteria: (i) published in peer-reviewed journals; (ii) employed IFA to detect antibodies to *O. tsutsugamushi* in human subjects; or (iii) published in English. Articles were excluded if they lacked accessible full texts, focussed on co-infections or animal studies without human relevance or did not involve serological testing with IFA.

### Data extraction and analysis

For the purposes of this review, if an article reported data from multiple distinct populations or combined findings from separate articles, each population or study was extracted and analysed as an independent sub-study. Data extraction was performed using a standardised form, capturing information on study design, IFA cut-off thresholds/seropositivity criteria, antigenic strains, and publication details. Geographic distribution, quality scores, and publication trends were also analysed. Diagnostic cut-off thresholds for both IgM and IgG were classified into standardised ranges to enable cross-study comparisons. This approach facilitated the identification of patterns and inconsistencies in serological interpretation across different contexts. Each study was categorised according to its design: (i) assay development or diagnostic validation, (ii) prospective recruitment such as a fever study, (iii) seroprevalence, (iv) diagnostic case-control and (v) case report studies. This classification provided insight into how study design might influence reported outcomes and the interpretation of assays. Additionally, the type and number of antigens used in the IFA assays were recorded, allowing for an assessment of how antigen composition may contribute to differences in assay sensitivity and specificity. Data analysis was conducted using RStudio (version 2024.04.02). Descriptive statistics summarised findings, and comparative analyses identified trends in diagnostic criteria and practices across regions and time periods.

### Quality assessment

The quality assessment scores of included articles were determined using a scoring system modified from the Standards for Reporting of Diagnostic Accuracy (STARD) checklist as described by Blacksell *and colleagues* [8,15]. Briefly, the evaluation focussed on whether: (i) the IFA methodology was cited correctly; (ii) the antigens used were specified; (iii) the antibody isotype(s)—IgM, IgG, or both—were indicated; (iv) at least one criterion for defining a positive result was described; and (v) a rationale was provided for the seropositivity threshold. Each criterion was scored as either 0 (not met) or 1 (met), resulting in a total quality score out of a maximum of 5. Variability in quality scores across publication years was evaluated using the Kruskal-Wallis test.

## Results

### Study selection

Our search identified 196 published articles. Of these, 84 were selected for inclusion in the final review (S1 Table and S1 Text). After accounting for articles containing multiple sub-studies, a total of 95 discrete datasets were analysed (Fig 1).

### General characteristics of the articles

**Fig 2:**
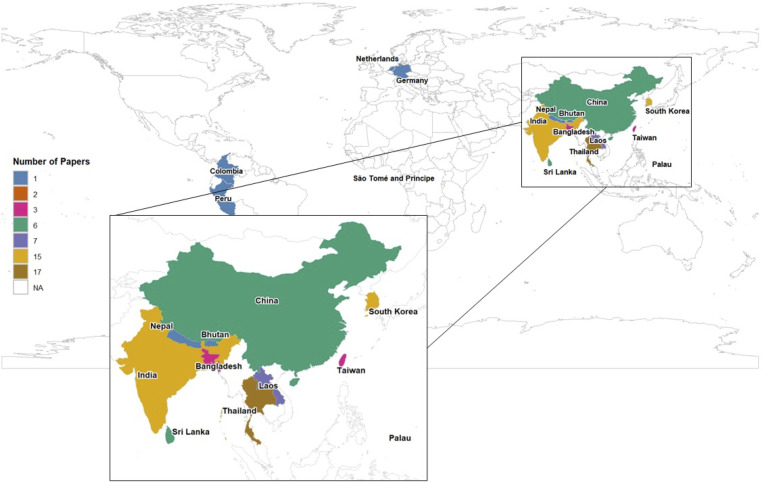
Geographic distribution and frequency of scrub typhus immunofluorescence assay articles. Base map shows country borders from Natural Earth (“Admin 0 – Countries”, medium resolution), accessed via the rnaturalearthdata R package (CC0 license; https://cran.r-project.org/package=rnaturalearthdata). The rnaturalearth interface package is MIT licensed (https://cran.r-project.org/package=rnaturalearth). Map data are in the public domain and freely available from Natural Earth (https://www.naturalearthdata.com).

We selected articles published from May 2005 to September 2024 to maintain a continuous record, following up on the original 2007 study. Among the 95 articles reviewed, 51 articles (53.7%) evaluated assay development or validation studies, 27 articles (28·4%) were prospective recruitment studies, 12 articles (12.6%) were cross-sectional/seroprevalence studies, 4 articles (4.2%) were case-control studies, and 1 article (1.1%) was a case report. Eighty-three articles were conducted across 16 countries, with the remaining 12 articles not specifying the location of the testing. The majority of studies were conducted in Thailand (17), India (15), and South Korea (15), with the remainder conducted in China, Germany, the Netherlands, Peru, Laos, Nepal, Bhutan, Bangladesh, Sri Lanka, Taiwan, Colombia, The Democratic Republic of São Tomé and Príncipe, and Republic of Palau (Fig 2).

### IFA cut-off criteria applied

**Fig 3:**
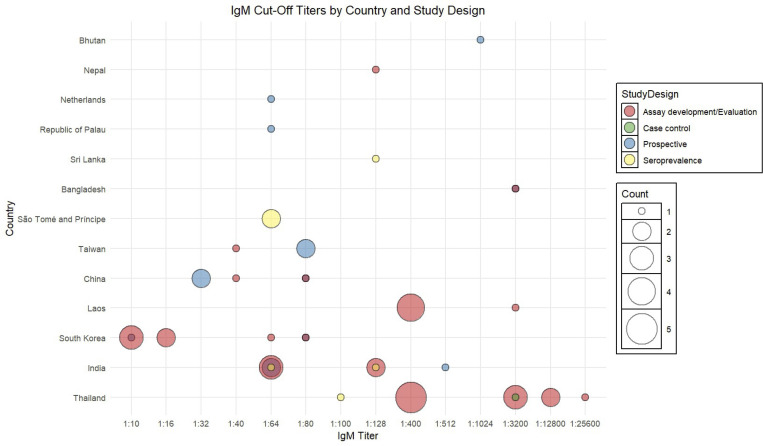
Variability in scrub typhus immunofluorescence assay IgM cut-off values across countries and study types.

**Fig 4:**
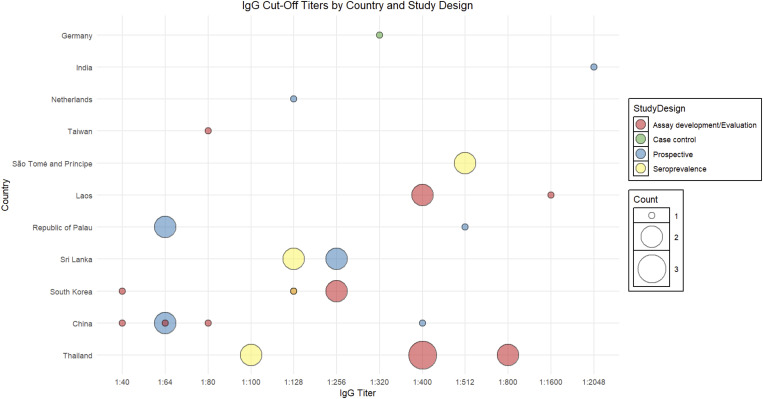
Variability in scrub typhus immunofluorescence assay IgG cut-off values across countries and study types.

IgM cut-off values were reported in 68 articles using 16 different thresholds to define seropositivity across 16 countries, ranging from 1:10 to 1:25,600 (Fig 3 and S2 Table). The most commonly applied were 1:64 (19.1%) and 1:400 (14.7%), followed by 1:80 (10.3%) and 1:3200 (7.4%). IgG cut-off thresholds for defining seropositivity, which range from 1:40 to 1:2,048, based on data from 40 articles conducted in 16 countries (Fig 4 and S3 Table). The most frequently reported threshold was 1:400 (15.0%), followed by 1:64 and 1:128 (each 12.5%), and 1:256 (10.0%). The 4-fold rise in antibody titre between acute and convalescent samples was described in 43 articles. However, it was often poorly defined, as a majority (81.4%) did not report the specific titres used (S3 Table).

### Articles cited for IFA methodology and cut-off criteria

Of the 95 studies analysed, only 39 (41.1%) cited at least one reference, with a maximum of four references, for the IFA methodology. A total of 34 articles were cited for the referenced methodologies, with the most frequently cited being the study by Bozeman *and colleagues* [16] and its subsequent modification by Robinson *and colleagues* [17]. Additionally, several articles by Blacksell *and colleagues* have been consistently cited as standard methodological references [7,8,18,19]. Only 37 articles (38.9%) justified the selected cut-off by referencing at least one prior article. A total of 28 distinct articles were referenced to justify seropositivity criteria (see S1 Table and S1 Text), with the articles by Brown *and colleagues* [20] and Lim *and colleagues* [21] being the most frequently cited.

### Assessment of diagnostic criteria by study type

Assay development studies (*n* = 55) made up the largest group and were primarily focussed on diagnostic assessment of in-house or commercial ELISA or PCR platforms. A striking observation was the wide variability in cut-off titres used for IgM detection, with values ranging from as low as 1:10 to as high as 1:25,600 (Fig 3 and S2 Table). The most commonly reported titres were 1:64, 1:80, and 1:400, with higher values often applied without a clear clinical rationale. IgG cut-offs, when specified, typically fell between 1:100 and 1:400 (Fig 4 and S3 Table).

Prospective recruitment studies (*n* = 21) provided the most clinically relevant evidence on IFA performance. These studies typically recruited patients with undifferentiated febrile illness and compared IFA or established reference standards, such as PCR or composite clinical case definitions. The focus was predominantly on IgM, reflecting its utility in identifying acute infections, though a subset of studies also included IgG, often through paired acute and convalescent samples. India featured prominently in this category, with studies demonstrating a broad use of titres (1:64, 1:80, and 1:400). Of the 21 prospective recruitment studies, 14 reported their cut-off titres for IgM, and most of these used titres of 1:64 (*n* = 4), 1:80 (*n* = 4), and 1:400 (*n* = 3).

Seroprevalence studies (*n* = 10) were primarily designed to estimate historical exposure to *O. tsutsugamushi* in defined populations. The cut-off titres reported in these studies ranged from 1:64 to 1:128 (IgM and IgG), with occasional use of higher thresholds such as 1:512 (IgG only). Two case reports included in the analysis described individual patients with confirmed scrub typhus, using results to support clinical diagnosis. Both reports described IgM and IgG findings, although only one specified the titre used (1:800). One of the reports was from Bangladesh, and the other did not specify a country. The two case-control studies compared results between confirmed scrub typhus patients and healthy or febrile controls. Both studies included IgM and IgG measurements. One case-control study reported using a notably high IgM cut-off of 1:3,200, while the other did not specify the threshold applied. One study was conducted in Thailand, and the second did not disclose the country.

### Geographic comparison of cut-offs

Thailand contributed one of the largest numbers of studies (*n* = 14), particularly in the category of assay development and reported IgM cut-off titres ranged widely from 1:128 up to 1:25,600 (Fig 3 and S2 Table). Notably, Thailand also performed studies with both very high cut-offs (e.g., 1:3,200, 1:12,800) and moderate cut-offs (e.g., 1:400), suggesting that selection was tailored to experimental purposes rather than diagnostic utility. India also featured prominently (*n* = 13), with a preference for mid-range IgM cut-off titres such as 1:64, 1:80, and 1:100. Studies from South Korea (*n* = 9) showed a striking tendency toward very low IgM cut-offs, including 1:10 and 1:16. These were mostly used in assay development studies and were accompanied by well-controlled sample sets. Lao studies (*n* = 5) included both diagnostic accuracy and assay development designs, commonly applying a cut-off of 1:400 for IgM. Chinese studies (*n* = 5) presented mixed use of IgM cut-offs, including values of 1:32, 1:40, and 1:80. Taiwanese studies (*n* = 3) generally applied lower-to-moderate cut-offs, including 1:40 and 1:80, with balanced consideration of both IgM and IgG. Bangladesh and Nepal each contributed one or two studies using higher titres (e.g., 1:3,200), though justification was sparse. São Tomé and Príncipe, Bhutan, Sri Lanka, and Palau had single or low-frequency studies that did not define or justify cut-offs clearly. Colombia, Germany, and Peru were included in the dataset but had no reported studies with specified cut-offs.

### Comparison of antigenic strains used for IFA

A comparison of the antigenic strains used in IFA across the included studies reveals considerable variation in strain selection, both in terms of strain combinations and geographic distribution (Table 1). The most commonly used antigenic panel comprised the “classic” trio of Kato, Karp, and Gilliam, reported in 29 articles (30.3%). These articles were predominantly conducted in Thailand (11 studies) and Laos (7), with additional representation from China, Taiwan, Sri Lanka, India, and Bangladesh. This combination remains the de facto standard in many regions of Asia, likely due to its historical use and commercial availability. The second most frequently employed panel was an expanded set including Kato, Karp, Gilliam, and Boryong, cited in 21 articles (22.1%). This configuration was widespread in India (9 articles) and South Korea (9 articles), where the inclusion of Boryong, a strain more representative of the South Korean *O. tsutsugamushi* landscape, may enhance diagnostic sensitivity. Studies from the Netherlands and Peru also used this combination, reflecting its adoption beyond endemic regions for research or imported case diagnoses. Several articles focussed on single-strain antigens, such as Karp alone (10.5%), used in studies from Sri Lanka, China, São Tomé and Príncipe, Colombia, Palau, and Germany, often for simplicity or specific strain prevalence. A smaller number of studies incorporated rare or more region-specific strains: for example, TA716 was used alongside Karp, Kato, and Gilliam in studies from Thailand, while *Candidatus* Orientia chuto appeared in studies from Nepal and Bhutan. Strains like Kangwon 87-61, Kuroki, and Kawasaki were limited to individual studies and were not widely adopted. The failure to disclose antigenic strain information in nearly a quarter of studies (23.2%) significantly hampers cross-study comparisons and limits reproducibility.

**Table 1:** A summary of antigenic strains identified in the selected articles was organised by the country where the indirect immunofluorescence assay was performed.

Antigen type	Number of articles (%)	Country (Number of Articles)
Kato, Karp, and Gilliam	29 (30.3)	Thailand (11), Laos (7), China (2), Taiwan (2), Sri Lanka (2), India (1), Bangladesh (2), Not Stated (2)
Kato, Karp, Gilliam, and Boryong	21 (22.1)	India (9), South Korea (9), Netherlands (1), Peru (1), Not stated (1)
Karp	10 (10.5)	China (2), Sri Lanka (2), The Democratic Republic of São Tomé and Príncipe (2), Republic of Palau (2), Colombia (1), Germany (1)
Karp, Kato, Gilliam, and TA716	3 (3.2)	Thailand (3)
Karp, Gilliam, Boryong	3 (3.2)	South Korea (3)
Kato, Karp, Gilliam, and *Candidatus* O. chuto	2 (2.1)	Nepal (1), Bhutan (1)
Gilliam, Karp, and Kangwon 87-61	2 (2.1)	South Korea (1), Not Stated (1)
Kato, Karp, Gilliam, Kuroki, and Kawasaki	1 (1.1)	Not Stated (1)
Gilliam	1 (1.1)	China (1)
Karp, Gilliam, and TA716	1 (1.1)	Not Stated (1)
Not Stated	22 (23.2)	India (5), Thailand (3), South Korea (2), Sri Lanka (2), China (1), Taiwan (1), Bangladesh (1), Republic of Palau (1), Not Stated (6)

### Quality assessment

**Fig 5:**
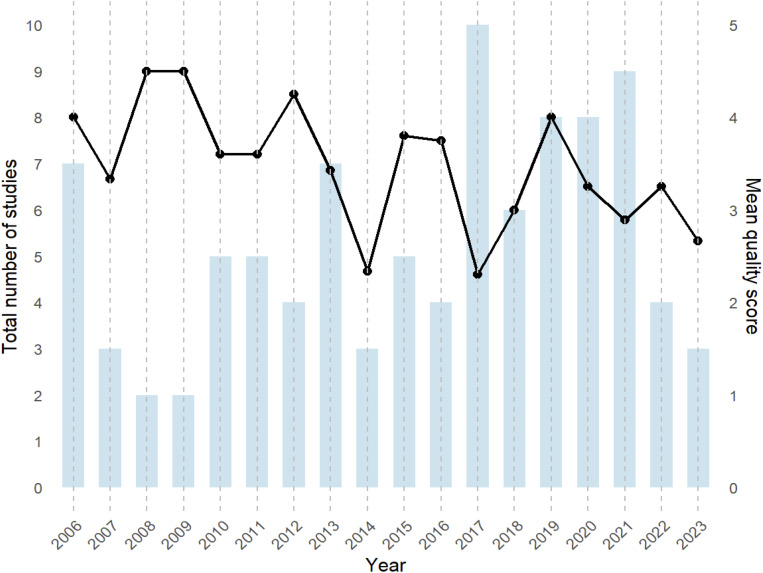
Distribution of published study numbers and mean quality scores from 2006 to 2023.

The total number of articles published annually exhibits considerable variation, with no consistent upward or downward trend (Fig 5). The highest publication volume occurred in 2017, with 11 articles, whereas 2007–2009, 2014–2016, and 2022–2023 recorded fewer publications, with fewer than 5 articles each (Fig 5). Visual inspection of annual means revealed noticeable fluctuations, with peaks in 2010, 2013, and 2018–2020 (mean scores approaching or exceeding 4) and lower scores in 2014 and 2023 (mean ~2.5). Despite fluctuations in annual publication volume and quality scores, no statistically significant improvement in methodological quality over time was observed (Kruskal–Wallis *H* = 24.5, *p* = 0.11). This suggests that variability in methodological reporting remains unresolved nearly two decades after it was first highlighted. Additionally, we performed a Spearman correlation test to assess whether the cut-off value was associated with study quality. The result showed a weak positive correlation between quality scores and cut-off values (*p* = 0.21), indicating that higher-quality studies reported higher cut-off values. Although not statistically significant (*p* = 0.095), the weak positive correlation suggests that higher quality studies may tend to report more conservative (higher) diagnostic cut-offs. This trend, if confirmed, could reflect greater methodological caution in avoiding false positives in endemic settings.

## Discussion

This scoping review identified marked heterogeneity in the diagnostic application of the IFA across studies published between May 2005 and September 2024. While ELISA-based serology is gaining popularity for diagnosing and researching scrub typhus [9,10], the IFA remains the preferred reference method due to its quantitative interpretability and adaptability for in-house use. However, widespread familiarity with the IFA, coupled with decades of incremental, uncoordinated methodological adaptations, has resulted in significant inconsistencies in interpretation, antigen selection, and reporting [8] that now require policy-level attention (see Table 2 for policy recommendations).

**Table 2:** Key policy priorities for standardising scrub typhus IFA and transitioning towards ELISA-based serology.

Key area	Current issue	Policy/action needed	Why it matters
**Cut-off standardisation**	IgM cut-offs range from 1:10 to 1:25,600; many not locally validated; STIC initially used ≥1:12,800 but later refined to ≥1:3,200 in Chiang Rai	Develop region-specific, evidence-based guidelines (e.g., adopt ≥1:3,200 for hyperendemic zones) validated via multi-site studies, referencing STIC refinements	Improves diagnostic comparability and reduces false positives/negatives; creates robust regional standards
**Paired vs. single sampling**	Many studies rely on single samples; fourfold rises often poorly reported	Mandate paired acute/convalescent sampling in reference labs and national surveillance; provide logistics support for follow-up	Captures dynamic antibody rises, improves diagnostic confidence and clinical relevance
**Antigen panel selection**	Antigen strain panels differ widely; 23% of studies did not report strains	Set minimal antigen panel requirements (e.g., Karp–Kato–Gilliam plus regionally important strains like Boryong); require full disclosure of panels	Ensures panels match circulating strains, improves sensitivity, allows cross-study comparison
**Quality and transparency of methods**	Only ~40% of studies cited methodology or justified cut-offs	Require STARD-like reporting standards for scrub typhus serology in journals, surveillance systems, and funding agreements	Builds reproducibility, credibility, and a reliable evidence base for future guidelines
**Alignment with ELISA and emerging platforms**	Poor correlation between IFA titres and ELISA optical densities; lack of transition guidance	Fund bridging studies mapping IFA titres (e.g., ≥1:3,200) to ELISA ODs; draft guidance for transitioning to validated ELISA platforms while retaining IFA as reference	Supports scalable testing, improves throughput, and harmonises data across platforms
**Regional validation of STIC**	Original STIC criteria (≥1:12,800 or fourfold rise) were stringent; later analyses suggest ≥1:3,200 is optimal in hyperendemic areas	Conduct pilot validation studies in regions with differing endemicity to adjust STIC thresholds; include Bayesian latent class models for improved accuracy	Tailors diagnostic algorithms to local epidemiology and improves policy applicability
**Global coordination and QC**	No international programme defining IFA standards or QC	Establish WHO/ASEAN-led working groups to issue consensus guidelines, distribute validated QC slides, and run external quality assurance schemes	Drives harmonisation and adoption across countries; ensures sustainable, comparable surveillance

### The future of the IFA as a “reference standard”

Our findings emphasise that the IFA’s status as a “gold standard” remains valid only if a harmonised framework is developed and adopted. The variation in IgM and IgG thresholds, the absence of minimum antigen panel requirements, and inconsistent reporting undermine its comparability. At the same time, ELISA platforms offer scalability and simplicity but lack calibrated correlation to IFA titres, creating a gap between the reference and operational tests. Direct comparison studies are limited, and discrepancies in cut-off titres between platforms further hinder comparability. Nevertheless, significant work has been performed in South Asia [14,22–24] and Southeast Asia [9,12,19] to better define regional cut-offs for in-house and commercial ELISAs. To future-proof serological surveillance, policy actions must include funded studies that map IFA titres to ELISA optical density (OD) values across diverse regions and standardise interpretation criteria. Without these actions, the IFA risks becoming a localised, in-house tool, while the broader diagnostic ecosystem fragments further.

### Cut-off heterogeneity and regional validation

Our review revealed striking heterogeneity in diagnostic cut-off thresholds for IgM and IgG, with at least 16 different IgM cut-offs reported, ranging from as low as 1:10 to as high as 1:25,600. Such wide variation reflects differences in background immunity, laboratory practices, institutional preferences, and study purposes. In hyperendemic settings, such as northern Thailand and Laos, higher IgM thresholds (≥1:400) were often employed to minimise false positives in populations with substantial background exposure. In contrast, some East Asian studies—particularly from South Korea—used very low cut-offs (1:10–1:16) to prioritise sensitivity in well-defined cohorts. South Asian studies often adopted mid-range thresholds (1:64–1:100), but these were rarely supported by explicit local validation data. For IgG, thresholds ranged from 1:40 to 1:2,048, again with considerable regional variation and limited justification. The adoption of mid-range thresholds without local validation has significant diagnostic consequences. Lowering the bar too far risks inflating seroprevalence estimates and exposing patients to unnecessary antimicrobial treatment. At the same time, overly conservative cut-offs may delay recognition of acute cases and compromise timely management. Both scenarios undermine the accuracy of surveillance data and the comparability of studies across regions. To address this, we recommend that South Asian programmes prioritise locally generated validation studies to establish thresholds that are both epidemiologically appropriate and operationally feasible. In turn, such evidence-based cut-offs should be embedded within national guidelines to ensure consistency across diagnostic platforms and settings.

A key finding is that while some regions have begun to select their cut-offs in local evidence, these efforts remain isolated rather than coordinated. The Scrub Typhus Infection Criteria (STIC) [25] developed in Chiang Rai, Thailand, illustrates a pathway toward standardisation. Initially, stringent thresholds (≥1:12,800 or a fourfold rise to that level) were proposed to ensure high specificity in research settings. However, subsequent analysis using Bayesian latent class models demonstrated that these thresholds achieved only moderate sensitivity, leading to refined cut-offs (≥1:3,200 or a fourfold rise to ≥1:3,200) that balance sensitivity (81.6%) and specificity (100%) in hyperendemic settings [21]. These findings have informed practices in neighbouring Laos, which adopted similar high thresholds due to comparable background immunity.

From a policy standpoint, these examples highlight the need to move beyond reliance on historical or imported thresholds. Ministries of health and regional bodies should support multi-site validation studies that use modern statistical approaches (e.g., latent class models) and robust reference standards to establish cut-offs that are epidemiologically appropriate for their populations. Once validated, these cut-offs should be codified in regional diagnostic guidelines and integrated into surveillance systems, with clear guidance on when and how to use paired sampling to confirm dynamic rises in titre. A good example is the nationwide study across 29 laboratories in India (aligned with the Indian Council of Medical Research) used IFA-confirmed cases and healthy controls to generate a ROC curve–derived pan-India IgM ELISA cut-off, which was then embedded into national diagnostic guidelines [26]. Such actions enable a more seamless alignment between IFA and ELISA platforms, as agreed cut-offs could then be used to calibrate new assays. Without this regional validation, countries risk continuing with thresholds that either over-call cases—leading to unnecessary treatments and inflated prevalence estimates—or underestimate the number cases, missing opportunities for timely management and control. A coordinated framework that recognises regional diversity but insists on evidence-based thresholds is essential to ensure that scrub typhus diagnostics are both locally relevant and globally comparable.

### Paired versus single sampling

One of the most consistent methodological challenges identified in this review was the widespread reliance on single serum samples for IFA interpretation, despite the well-established principle that scrub typhus diagnosis is most robust when based on a dynamic change in antibody levels between acute and convalescent samples. Ideally, paired sampling—collecting a second sample 7–14 days after illness onset—allows for the demonstration of a fourfold or greater rise in titre, which remains the most reliable criterion for confirming a recent infection [8]. However, our findings show that many studies either did not collect convalescent samples or failed to report whether paired serology was performed. In some cases, single high cut-offs were used as a proxy, particularly in hyperendemic settings. Still, this approach can lead to both false positives (due to background immunity) and false negatives (if titres have not yet peaked). This heterogeneity not only undermines diagnostic certainty in clinical care but also compromises the comparability of surveillance data across regions.

### Justification for methodologies and diagnostic cut-offs

Our study highlights the variation and reporting quality of the IFA methodology itself. The frequent citation of Bozeman *and colleagues* [16] and Robinson *and colleagues* [17] underscores the reliance on older foundational work. In contrast, the increasing citation of Blacksell *and colleagues* [27–29] and the application of the STIC [21,25] reflect a growing influence of Southeast Asian research centres in shaping diagnostic norms.

### Antigenic strain composition

The geographic distribution of *O. tsutsugamushi* strains shows clear regional patterns, which have important implications for diagnostics and vaccine development [30]. Karp is the most widely distributed strain in Asia [31–34] and northern Australia [30], while Gilliam are also commonly found, particularly in India [33,35], Thailand, South Korea [36], and China [37,38]. Boryong is confined mainly to South Korea [36,39]. Kato-like strains have been reported in India [35,40] and South Korea [36]. TA716 and TA763 appear to be more restricted to Southeast Asia, particularly Thailand [31,32], although they have recently been described in Nagaland, India [41]. These regional differences in strain prevalence have directly influenced antigen selection in diagnostic assays, with Karp-Kato-Gilliam combinations commonly used across diverse settings and Boryong frequently included in Korean studies. While there is substantial serological cross-reactivity between strains (i.e., prototype Karp, Gilliam, and TA strains [30,32,42], evident antigenic disparities have been demonstrated with the Kato strain [30] which has significant diagnostic implications. This antigenic diversity highlights the need for region-specific surveillance and diagnostic strategies that take into account the circulating strain profiles. In this study, we have found that the classic Kato–Karp–Gilliam panel remains the most commonly used antigen combination, particularly in Southeast Asian studies; its continued dominance likely reflects historical convention rather than contemporary epidemiological relevance. The inclusion of Boryong in nearly a quarter of studies, especially those from South Korea, suggests a regional adaptation toward greater strain coverage, although the inclusion for the Indian studies is puzzling. However, the reliance on single-strain panels and the absence of antigen strain details in nearly a quarter of the studies highlight potential gaps in antigenic coverage. Therefore, failure to disclose or standardise strain panels undermines diagnostic comparability and limits the utility of the assay in emerging endemic areas. Furthermore, variability in antigen preparation—especially with in-house slides—may result in batch-to-batch differences that impact sensitivity and reproducibility. Establishing minimal standards for antigen quality control and validation should be an integral part of standardisation efforts.

## Conclusion

In summary, this review underscores that achieving meaningful standardisation of scrub typhus serology requires more than technical refinements—it requires coordinated health policy action. Region-specific cut-offs validated through multi-site studies (building on STIC refinements), mandated paired sampling, standardised antigen panels, transparent reporting, and harmonisation between IFA and ELISA are all essential. A global or regional framework, led by WHO and partner organisations, could drive these actions and transform the current patchwork of practices into a robust, scalable diagnostic system capable of supporting both clinical management and surveillance in endemic and emerging areas.

Looking ahead, the development of evidence-based serological cut-offs will require not only laboratory validation but also improved understanding of the regional endemic disease background, including reliable incidence and prevalence data. These epidemiological foundations are essential to ensure that diagnostic thresholds are both clinically meaningful and context-appropriate. Building such a framework will depend on collaborative networks of clinical, diagnostic, and epidemiological experts, as well as the sustained experience of regional laboratories. With coordinated effort, these data-driven approaches can provide a stronger basis for harmonised guidelines and improved patient care across endemic areas.

## Supporting information

S1 Table Summary of studies. (DOCX)

S2 Table Summary of single-titre diagnostic cut-offs of IgM reported in the selected articles. (DOCX)

S3 Table Summary of single-titre diagnostic cut-offs of IgG, total antibody and Four-fold increase titre reported in the selected articles. (DOCX)

S1 Text References for S1 Table. (DOCX)
