# Supplementary material for: Diagnostic heterogeneity in scrub typhus serology: A scoping review of IFA thresholds and regional standardisation needs (2005–2024)
Source: PLoS Negl Trop Dis. 2025 Oct 22;19(10):e0013540. doi: 10.1371/journal.pntd.0013540 (PMC12543141; doi:10.1371/journal.pntd.0013540)
Supplement: S2 Table — (DOCX) [file pntd.0013540.s002.docx]

# **S2 Table. Summary of single-titer diagnostic cut-offs of IgM reported in the selected articles.**

|  | **IgM cut-off titer** | | | | | | | | | | | | | | | | | |
| --- | --- | --- | --- | --- | --- | --- | --- | --- | --- | --- | --- | --- | --- | --- | --- | --- | --- | --- |
|  | 1:10 | 1:16 | 1:32 | 1:40 | 1:64 | 1:80 | 1:100 | 1:128 | 1:160 | 1:400 | 1:512 | 1:800 | 1:1024 | 1:3200 | 1:12800 | 1:25600 | NS*^1^* | **Total (%)** |
| **Study number, n (%)** | 5 (7.4) | 2 (2.9) | 2 (2.9) | 2 (2.9) | 13 (19.1) | 7 (10.3) | 1 (1.5) | 5 (7.4) | 1 (1.5) | 10 (14.7) | 1 (1.5) | 1 (1.5) | 1 (1.5) | 7 (10.3) | 2 (2.9) | 2 (2.9) | 6 (8.8) | **68** |
| **Study design** |  |  |  |  |  |  |  |  |  |  |  |  |  |  |  |  |  |  |
| Assay development | 4 | 2 | ... | 2 | 6 | 3 | ... | 3 | 1 | 9 | ... | ... | ... | 5 | 2 | 2 | 2 | 41 (60.2) |
| Prospective recruitment | 1 | ... | 2 | ... | 4 | 4 | ... | ... | ... | 1 | 1 | ... | 1 | 1 | ... | ... | 3 | 18 (26.5) |
| Seroprevalence | ... | ... | ... | ... | 3 | ... | 1 | 2 | ... | ... | ... | ... | ... | ... | ... | … | 1 | 7 (10.3) |
| Case report | ... | ... | ... | ... | ... | ... | ... | ... | ... | ... | ... | 1 | ... | ... | ... | ... | ... | 1 (1.5) |
| Case-control | ... | ... | ... | ... | ... | ... | ... | ... | ... | ... | ... | ... | ... | 1 | ... | ... | ... | 1 (1.5) |
| **Country** |  |  |  |  |  |  |  |  |  |  |  |  |  |  |  |  |  |  |
| Thailand | ... | ... | ... | ... | ... | ... | 1 | ... | … | 5 | ... | ... | ... | 4 | 2 | 1 | 1 | 14 (20.5) |
| India | ... | ... | ... | … | 6 | ... | ... | 3 | ... | ... | 1 | ... | ... | ... | ... | ... | 3 | 13 (19.1) |
| South Korea | 4 | 2 | ... | ... | 1 | 2 | ... | ... | ... | ... | ... | ... | ... | ... | ... | ... | ... | 9 (13.2) |
| China | ... | ... | 2 | 1 | ... | 2 | ... | ... | ... | ... | ... | ... | ... | ... | ... | ... | ... | 5 (7.4) |
| Laos | ... | ... | ... | ... | ... | ... | ... | ... | ... | 4 | ... | ... | ... | 1 | ... | ... | ... | 5 (7.4) |
| Taiwan | ... | ... | ... | 1 | ... | 2 | ... | ... | ... | ... | ... | ... | ... | ... | ... | ... | ... | 3 (4.4) |
| The Democratic Republic of São Tomé and Príncipe | ... | ... | ... | ... | 2 | ... | ... | ... | ... | ... | ... | ... | ... | ... | ... | ... | ... | 2 (2.9) |
| Bangladesh | ... | ... | ... | ... | ... | ... | ... | ... | ... | ... | ... | ... | ... | 2 | ... | ... | ... | 2 (2.9) |
| Sri Lanka | ... | ... | ... | ... | ... | ... | ... | 1 | ... | ... | ... | ... | ... | ... | ... | ... | ... | 1 (1.5) |
| Nepal | ... | ... | ... | ... | ... | ... | ... | 1 | ... | ... | ... | ... | ... | ... | ... | ... | ... | 1 (1.5) |
| Netherlands | ... | ... | ... | ... | 1 | ... | ... | ... | ... | ... | ... | ... | ... | ... | ... | ... | ... | 1 (1.5) |
| Bhutan | ... | ... | ... | ... |  | ... | ... | ... | ... | ... | ... | ... | 1 | ... | ... | ... | ... | 1 (1.5) |
| Republic of Palau | ... | ... |  | ... | 1 | ... | ... | ... | ... | ... | ... | ... | ... | ... | ... | ... | ... | 1 (1.5) |
| Not stated^2^ | 1 | ... | ... | ... | 2 | 1 | ... | ... | 1 | 1 | ... | 1 | ... | ... | ... | 1 | 2 | 10 (14.7) |
| ^1^ The study does not specify a cut-off value  ^2^ The study does not specify a country | | | | | | | | | | | | | | | | | | |
