# Supplementary material for: Diagnostic heterogeneity in scrub typhus serology: A scoping review of IFA thresholds and regional standardisation needs (2005–2024)
Source: PLoS Negl Trop Dis. 2025 Oct 22;19(10):e0013540. doi: 10.1371/journal.pntd.0013540 (PMC12543141; doi:10.1371/journal.pntd.0013540)
Supplement: S3 Table — (DOCX) [file pntd.0013540.s003.docx]

# **S3 Table. Summary of single-titer diagnostic cut-offs of IgG, total antibody and Four-fold increase titer reported in the selected articles**

|  | **IgG cut-off titer** | | | | | | | | | | | | | |  | **Total antibody^3^ titer** | | | | | **Four-fold titer increase** | | | | |
| --- | --- | --- | --- | --- | --- | --- | --- | --- | --- | --- | --- | --- | --- | --- | --- | --- | --- | --- | --- | --- | --- | --- | --- | --- | --- |
|  | 1:40 | 1:64 | 1:80 | 1:100 | 1:128 | 1:256 | 1:320 | 1:400 | 1:512 | 1:800 | 1:1600 | 1:2048 | NS^1^ | **Total (%)** | 1:40 | | 1:320 | 1:400 | NS^1^ | **Total (%)** | 1:200 | 1:3200 | NS^1^ | | **Total (%)** |
| **Study number, n (%)** | 3 (7.5) | 5 (12.5) | 2 (5.0) | 2 (5.0) | 5 (12.5) | 4 (10.0) | 1 (2.5) | 6 (15.0) | 3 (7.5) | 2 (5.0) | 1 (2.5) | 1 (2.5) | 5 (12.5) | **40** | 2 (33.3) | | 1 (16.7) | 1 (16.7) | 2 (33.3) | **6** | 1 (2.3) | 7 (16.3) | 35 (81.4) | | **43** |
| **Study design** |  |  |  |  |  |  |  |  |  |  |  |  |  |  |  | |  |  |  |  |  |  | |  |  |
| Assay development | 3 | 1 | 2 | ... | 1 | 2 | ... | 5 | ... | 2 | 1 | ... | ... | 17 (42.5) | 2 | | ... | ... | ... | 2 (33.3) | ... | 5 | | 23 | 28 (65.1) |
| Prospective recruitment | ... | 4 | ... | ... | 1 | 2 | ... | 1 | 1 | ... | ... | 1 | 3 | 13 (32.5) | ... | | ... | 1 | 2 | 3 (50.0) | 1 | 1 | | 10 | 12 (27.9) |
| Seroprevalence | ... | ... | ... | 2 | 3 | ... | ... | ... | 2 | ... | ... | ... | 2 | 9 (22.5) | ... | | ... | ... | ... | 0 | ... | ... | | 1 | 1 (2.3) |
| Case-control | ... | ... | ... | ... | ... | ... | 1 | ... | ... | ... | ... | ... | ... | 1 (2.5) | ... | | 1 | ... | ... | 1 (16.7) | ... | 1 | | 1 | 2 (4.7) |
| **Country** |  |  |  |  |  |  |  |  |  |  |  |  |  |  |  | |  |  |  |  |  |  | |  |  |
| Thailand | ... | ... | ... | 2 | ... | ... | ... | 3 | ... | 2 | ... | ... | 1 | 8 (20.0) | ... | | ... | 1 | ... | 1 (16.7) | 1 | 4 | | 9 | 14 (32.5) |
| China | 1 | 3 | 1 | ... | ... | ... | ... | 1 | ... | ... | ... | ... | ... | 6 (15.0) | ... | | ... | ... | ... | 0 | ... | ... | | 2 | 2 (4.7) |
| South Korea | 1 | ... | ... | ... | 2 | 2 | ... | ... | ... | ... | ... | ... | ... | 5 (12.5) | 2 | | ... | ... | ... | 2 (33.3) | ... | ... | | 9 | 9 (20.9) |
| Sri Lanka | ... | ... | ... | ... | 2 | 2 | ... | ... | ... | ... | ... |  | 1 | 5 (12.5) | ... | | ... | ... | ... | 0 | ... | ... | | ... | 0 |
| India | ... |  | ... | ... | ... | ... | ... | ... |  | ... | … | 1 | 2 | 3 (7.5) | ... | | ... | ... | 1 | 1 (16.7) | ... | ... | | 2 | 2 (4.7) |
| Laos | ... | ... |  | ... | ... | ... | ... | 2 | ... | ... | 1 | ... | ... | 3 (7.5) | ... | | ... | ... | ... | 0 | ... | 1 | | 4 | 5 (11.6) |
| Republic of Palau | ... | 2 | ... | ... | ... | ... | ... | ... | 1 | ... | ... | ... | ... | 3 (7.5) | ... | | ... | ... | ... | 0 | ... | ... | | ... | 0 |
| The Democratic Republic of São Tomé and Príncipe | ... | ... | ... | ... | ... | ... | ... | ... | 2 | ... | ... | ... | ... | 2 (5.0) | ... | | ... | ... | ... | 0 | ... | ... | | ... | 0 |
| Colombia | ... | ... | ... | ... |  | ... | ... | ... | ... | ... | ... | ... | 1 | 1 (2.5) | ... | | ... | ... | ... | 0 | ... | ... | | ... | 0 |
| Taiwan | ... | ... | 1 | ... | ... | ... | ... | ... | ... | ... | ... | ... | ... | 1 (2.5) | ... | | ... | ... | 1 | 1 (16.7) | ... | ... | | 3 | 3 (7.0) |
| Germany | ... | … | ... | ... | ... | ... | 1 | ... | ... | ... | ... | ... | ... | 1 (2.5) | ... | | 1 | ... | ... | 1 (16.7) | ... | ... | | ... | 0 |
| Netherlands | ... | ... | ... | ... | 1 | ... | ... | ... | ... | ... | ... | ... | ... | 1 (2.5) | ... | | ... | ... | ... | 0 | ... | ... | | 1 | 1 (2.3) |
| Bangladesh | ... | ... | ... | ... | ... | ... | ... | ... | ... | ... | ... | ... | ... | 0 | ... | | ... | ... | ... | 0 | ... | 2 | | ... | 2 (4.7) |
| Not stated^2^ | 1 | ... | ... | ... | ... | ... | ... | ... | ... | ... | ... | ... | ... | 1 (2.5) | ... | | ... | ... | ... | 0 | ... | ... | | 5 | 5 (11.6) |
| ^1^The study does not specify a cut-off value  ^2^ The study does not specify a country  ^3^ Total antibody =IgM + IgG + IgA antibody isotypes | | | | | | | | | | | | | | | | | | | | | | | | | |
