# Supplementary material for: Diagnostic heterogeneity in scrub typhus serology: A scoping review of IFA thresholds and regional standardisation needs (2005–2024)
Source: PLoS Negl Trop Dis. 2025 Oct 22;19(10):e0013540. doi: 10.1371/journal.pntd.0013540 (PMC12543141; doi:10.1371/journal.pntd.0013540)
Supplement: S1 Text — (DOCX) [file pntd.0013540.s004.docx]

# **S1 Text - References for S1 Table.**

1. Kim DM, Kim HL, Park CY, Yang TY, Lee JH, Yang JT, et al. Clinical usefulness of eschar polymerase chain reaction for the diagnosis of scrub typhus: a prospective study. Clin Infect Dis. 2006;43(10):1296-300. Epub 2006/10/20. doi: 10.1086/508464. PubMed PMID: 17051495.

2. Kim DM, Yun NR, Yang TY, Lee JH, Yang JT, Shim SK, et al. Usefulness of nested PCR for the diagnosis of scrub typhus in clinical practice: A prospective study. Am J Trop Med Hyg. 2006;75(3):542-5. Epub 2006/09/14. PubMed PMID: 16968938.

3. Sonthayanon P, Chierakul W, Wuthiekanun V, Blacksell SD, Pimda K, Suputtamongkol Y, et al. Rapid diagnosis of scrub typhus in rural Thailand using polymerase chain reaction. Am J Trop Med Hyg. 2006;75(6):1099-102. PubMed PMID: 17172374.

4. Demma LJ, McQuiston JH, Nicholson WL, Murphy SM, Marumoto P, Sengebau-Kingzio M, et al. Scrub typhus, Republic of Palau. Emerg Infect Dis. 2006;12(2):290-5. Epub 2006/02/24. doi: 10.3201/eid1202.050967. PubMed PMID: 16494757; PubMed Central PMCID: PMCPMC3373099.

5. Cao M, Guo H, Tang T, Wang C, Li X, Pan X, et al. Preparation of recombinant antigen of O. *tsutsugamushi*  Ptan strain and development of rapid diagnostic reagent for scrub typhus. Am J Trop Med Hyg. 2007;76(3):553-8. Epub 2007/03/16. PubMed PMID: 17360883.

6. Kim DM, Won KJ, Park CY, Yu KD, Kim HS, Yang TY, et al. Distribution of eschars on the body of scrub typhus patients: a prospective study. Am J Trop Med Hyg. 2007;76(5):806-9. Epub 2007/05/10. PubMed PMID: 17488895.

7. Chanta C, Triratanapa K, Ratanasirichup P, Mahaprom W. Hepatic dysfunction in pediatric scrub typhus: role of liver function test in diagnosis and marker of disease severity. J Med Assoc Thai. 2007;90(11):2366-9. Epub 2008/01/10. PubMed PMID: 18181321.

8. Paris DH, Blacksell SD, Stenos J, Graves SR, Unsworth NB, Phetsouvanh R, et al. Real-time multiplex PCR assay for detection and differentiation of rickettsiae and *Orientia*e. Trans R Soc Trop Med Hyg. 2008;102(2):186-93. doi: 10.1016/j.trstmh.2007.11.001. PubMed PMID: 18093627.

9. Kim DM, Lee YM, Back JH, Yang TY, Lee JH, Song HJ, et al. A serosurvey of *Orientia* *tsutsugamushi*  from patients with scrub typhus. Clin Microbiol Infect. 2010;16(5):447-51. Epub 2009/09/26. doi: 10.1111/j.1469-0691.2009.02865.x. PubMed PMID: 19778303.

10. Kim DM, Park CJ, Lim SC, Park KH, Jang WJ, Lee SH. Diagnosis of scrub typhus by immunohistochemical staining of *Orientia* *tsutsugamushi*  in cutaneous lesions. Am J Clin Pathol. 2008;130(4):543-51. Epub 2008/09/17. doi: 10.1309/x17hnnjkmyght4hp. PubMed PMID: 18794046.

11. Phetsouvanh R, Blacksell SD, Jenjaroen K, Day NP, Newton PN. Comparison of indirect immunofluorescence assays for diagnosis of scrub typhus and murine typhus using venous blood and finger prick filter paper blood spots. Am J Trop Med Hyg. 2009;80(5):837-40. PubMed PMID: 19407134.

12. Liu YX, Feng D, Suo JJ, Xing YB, Liu G, Liu LH, et al. Clinical characteristics of the autumn-winter type scrub typhus cases in south of Shandong province, northern China. BMC Infect Dis. 2009;9:82. Epub 2009/06/06. doi: 10.1186/1471-2334-9-82. PubMed PMID: 19493361; PubMed Central PMCID: PMCPMC2703643.

13. Blacksell SD, Jenjaroen K, Phetsouvanh R, Tanganuchitcharnchai A, Phouminh P, Phongmany S, et al. Accuracy of rapid IgM-based immunochromatographic and immunoblot assays for diagnosis of acute scrub typhus and murine typhus infections in Laos. Am J Trop Med Hyg. 2010;83(2):365-9. doi: 10.4269/ajtmh.2010.09-0534. PubMed PMID: 20682883; PubMed Central PMCID: PMCPMC2911186.

14. Zhang S, Song H, Liu Y, Li Q, Wang Y, Wu J, et al. Scrub typhus in previously unrecognized areas of endemicity in China. J Clin Microbiol. 2010;48(4):1241-4. Epub 2010/02/05. doi: 10.1128/jcm.01784-09. PubMed PMID: 20129967; PubMed Central PMCID: PMCPMC2849583.

15. Jeong HW, Choi YK, Baek YH, Seong MH. Phylogenetic analysis of the 56-kDa type-specific protein genes of *Orientia* *tsutsugamushi*  in Central Korea. J Korean Med Sci. 2012;27(11):1315-9. Epub 2012/11/21. doi: 10.3346/jkms.2012.27.11.1315. PubMed PMID: 23166411; PubMed Central PMCID: PMCPMC3492664.

16. Lijuan Z, Si H, Yuming J, Liang L, Xuemei L, Lianying L, et al. A rapid, sensitive and reliable diagnostic test for scrub typhus in China. Indian J Med Microbiol. 2011;29(4):368-71. Epub 2011/11/29. doi: 10.4103/0255-0857.90166. PubMed PMID: 22120796.

17. Zhang L, He S, Wang S, Yu H, Li X, Zhang D, et al. Comparison of a rapid diagnostic test and microimmunofluorescence assay for detecting antibody to *Orientia* *tsutsugamushi*  in scrub typhus patients in China. Asian Pac J Trop Med. 2011;4(8):666-8. Epub 2011/09/15. doi: 10.1016/s1995-7645(11)60169-7. PubMed PMID: 21914549.

18. Paris DH, Blacksell SD, Nawtaisong P, Jenjaroen K, Teeraratkul A, Chierakul W, et al. Diagnostic accuracy of a loop-mediated isothermal PCR assay for detection of *Orientia* *tsutsugamushi*  during acute Scrub Typhus infection. PLoS Negl Trop Dis. 2011;5(9):e1307. doi: 10.1371/journal.pntd.0001307. PubMed PMID: 21931873; PubMed Central PMCID: PMCPMC3172190.

19. Liyanapathirana VC, Thevanesam V. Seroepidemiology of rickettsioses in Sri Lanka: a patient based study. BMC Infect Dis. 2011;11:328. Epub 2011/11/29. doi: 10.1186/1471-2334-11-328. PubMed PMID: 22118601; PubMed Central PMCID: PMCPMC3248378.

20. Ruang-Areerate T, Jeamwattanalert P, Rodkvamtook W, Richards AL, Sunyakumthorn P, Gaywee J. Genotype diversity and distribution of *Orientia* *tsutsugamushi*  causing scrub typhus in Thailand. J Clin Microbiol. 2011;49(7):2584-9. Epub 2011/05/20. doi: 10.1128/jcm.00355-11. PubMed PMID: 21593255; PubMed Central PMCID: PMCPMC3147819.

21. Premaratna R, Weerasinghe S, Ranaweera A, Chandrasena TG, Bandara NW, Dasch GA, et al. Clinically helpful rickettsial disease diagnostic IgG titers in relation to duration of illness in an endemic setting in Sri Lanka. BMC Res Notes. 2012;5:662. Epub 2012/12/04. doi: 10.1186/1756-0500-5-662. PubMed PMID: 23198969; PubMed Central PMCID: PMCPMC3536648.

22. Silpasakorn S, Srisamut N, Ekpo P, Zhang Z, Chao CC, Ching WM, et al. Development of new, broadly reactive, rapid IgG and IgM lateral flow assays for diagnosis of scrub typhus. Am J Trop Med Hyg. 2012;87(1):148-52. Epub 2012/07/06. doi: 10.4269/ajtmh.2012.11-0583. PubMed PMID: 22764306; PubMed Central PMCID: PMCPMC3391040 Government.

23. Paris DH, Phetsouvanh R, Tanganuchitcharnchai A, Jones M, Jenjaroen K, Vongsouvath M, et al. *Orientia* *tsutsugamushi*  in human scrub typhus eschars shows tropism for dendritic cells and monocytes rather than endothelium. PLoS Negl Trop Dis. 2012;6(1):e1466. doi: 10.1371/journal.pntd.0001466. PubMed PMID: 22253938; PubMed Central PMCID: PMCPMC3254662.

24. Silpasakorn S, Waywa D, Hoontrakul S, Suttinont C, Losuwanaluk K, Suputtamongkol Y. Performance of SD Bioline *Tsutsugamushi*  assays for the diagnosis of scrub typhus in Thailand. J Med Assoc Thai. 2012;95 Suppl 2:S18-22. Epub 2012/05/12. PubMed PMID: 22574525.

25. Watthanaworawit W, Turner P, Turner C, Tanganuchitcharnchai A, Richards AL, Bourzac KM, et al. A prospective evaluation of real-time PCR assays for the detection of *Orientia* *tsutsugamushi*  and Rickettsia spp. for early diagnosis of rickettsial infections during the acute phase of undifferentiated febrile illness. Am J Trop Med Hyg. 2013;89(2):308-10. Epub 2013/06/05. doi: 10.4269/ajtmh.12-0600. PubMed PMID: 23732256; PubMed Central PMCID: PMCPMC3741253 Society for Microbiology 112th General Meeting (ASM 2012) in San Francisco, California, June 16–19, 2012.

26. Lee K-D, Moon C, Oh WS, Sohn KM, Kim B-N. Diagnosis of scrub typhus: introduction of the immunochromatographic test in Korea. The Korean Journal of Internal Medicine. 2014;29:253 - 5.

27. Li W, Dou X, Zhang L, Lyu Y, Du Z, Tian L, et al. Laboratory diagnosis and genotype identification of scrub typhus from Pinggu district, Beijing, 2008 and 2010. Am J Trop Med Hyg. 2013;89(1):123-9. Epub 2013/05/30. doi: 10.4269/ajtmh.12-0728. PubMed PMID: 23716411; PubMed Central PMCID: PMCPMC3748468.

28. Wang YC, Chen PC, Lee KF, Wu YC, Chiu CH. Scrub typhus cases in a teaching hospital in Penghu, Taiwan, 2006-2010. Vector Borne Zoonotic Dis. 2013;13(3):154-9. Epub 2013/02/21. doi: 10.1089/vbz.2012.1059. PubMed PMID: 23421889.

29. Phetsouvanh R, Thojaikong T, Phoumin P, Sibounheuang B, Phommasone K, Chansamouth V, et al. Inter- and intra-operator variability in the reading of indirect immunofluorescence assays for the serological diagnosis of scrub typhus and murine typhus. Am J Trop Med Hyg. 2013;88(5):932-6. Epub 2013/03/13. doi: 10.4269/ajtmh.12-0325. PubMed PMID: 23478577; PubMed Central PMCID: PMCPMC3752761.

30. Kularatne SA, Rajapakse RP, Wickramasinghe WM, Nanayakkara DM, Budagoda SS, Weerakoon KG, et al. Rickettsioses in the central hills of Sri Lanka: serological evidence of increasing burden of spotted fever group. Int J Infect Dis. 2013;17(11):e988-92. Epub 2013/07/23. doi: 10.1016/j.ijid.2013.05.014. PubMed PMID: 23871280.

31. Kim YJ, Yeo SJ, Park SJ, Woo YJ, Kim MW, Kim SH, et al. Improvement of the diagnostic sensitivity of scrub typhus using a mixture of recombinant antigens derived from *Orientia* *tsutsugamushi*  serotypes. J Korean Med Sci. 2013;28(5):672-9. Epub 2013/05/17. doi: 10.3346/jkms.2013.28.5.672. PubMed PMID: 23678257; PubMed Central PMCID: PMCPMC3653078.

32. Lai CH, Chang LL, Lin JN, Tsai KH, Hung YC, Kuo LL, et al. Human spotted fever group rickettsioses are underappreciated in southern Taiwan, particularly for the species closely-related to *Rickettsia felis*. PLoS One. 2014;9(4):e95810. Epub 2014/04/24. doi: 10.1371/journal.pone.0095810. PubMed PMID: 24755560; PubMed Central PMCID: PMCPMC3995941.

33. Premaratna R, Ariyaratna N, Attanayake C, Bandara W, Chandrasena N, de Silva HJ. Rickettsial infection among military personnel deployed in Northern Sri Lanka. BMC Infect Dis. 2014;14:3864. Epub 2014/12/21. doi: 10.1186/s12879-014-0688-8. PubMed PMID: 25527099; PubMed Central PMCID: PMCPMC4335424.

34. Koraluru M, Bairy I, Varma M, Vidyasagar S. Diagnostic validation of selected serological tests for detecting scrub typhus. Microbiol Immunol. 2015;59(7):371-4. Epub 2015/05/27. doi: 10.1111/1348-0421.12268. PubMed PMID: 26011315.

35. Watthanaworawit W, Turner P, Turner C, Tanganuchitcharnchai A, Jintaworn S, Hanboonkunupakarn B, et al. Diagnostic Accuracy Assessment of Immunochromatographic Tests for the Rapid Detection of Antibodies Against *Orientia* *tsutsugamushi*  Using Paired Acute and Convalescent Specimens. Am J Trop Med Hyg. 2015;93(6):1168-71. Epub 2015/10/16. doi: 10.4269/ajtmh.15-0435. PubMed PMID: 26458778; PubMed Central PMCID: PMCPMC4674230.

36. Rodkvamtook W, Zhang Z, Chao CC, Huber E, Bodhidatta D, Gaywee J, et al. Dot-ELISA Rapid Test Using Recombinant 56-kDa Protein Antigens for Serodiagnosis of Scrub Typhus. Am J Trop Med Hyg. 2015;92(5):967-71. Epub 2015/03/25. doi: 10.4269/ajtmh.14-0627. PubMed PMID: 25802430; PubMed Central PMCID: PMCPMC4426586.

37. Blacksell SD, Tanganuchitcharnchai A, Nawtaisong P, Kantipong P, Laongnualpanich A, Day NP, et al. Diagnostic Accuracy of the InBios Scrub Typhus Detect Enzyme-Linked Immunoassay for the Detection of IgM Antibodies in Northern Thailand. Clin Vaccine Immunol. 2016;23(2):148-54. Epub 2015/12/15. doi: 10.1128/CVI.00553-15. PubMed PMID: 26656118; PubMed Central PMCID: PMCPMC4744921.

38. Lim C, Blacksell SD, Laongnualpanich A, Kantipong P, Day NP, Paris DH, et al. Optimal Cutoff Titers for Indirect Immunofluorescence Assay for Diagnosis of Scrub Typhus. J Clin Microbiol. 2015;53(11):3663-6. doi: 10.1128/JCM.01680-15. PubMed PMID: 26354819.

39. Kim YJ, Park S, Premaratna R, Selvaraj S, Park SJ, Kim S, et al. Clinical Evaluation of Rapid Diagnostic Test Kit for Scrub Typhus with Improved Performance. J Korean Med Sci. 2016;31(8):1190-6. Epub 2016/08/02. doi: 10.3346/jkms.2016.31.8.1190. PubMed PMID: 27478327; PubMed Central PMCID: PMCPMC4951546.

40. Gupta N, Chaudhry R, Kabra SK, Lodha R, Mirdha BR, Das BK, et al. Comparative Evaluation of Serological and Molecular Methods for the Diagnosis of Scrub Typhus in Indian Settings. Jpn J Infect Dis. 2017;70(2):221-2. Epub 2016/09/02. doi: 10.7883/yoken.JJID.2016.139. PubMed PMID: 27580576.

41. Gupta N, Chaudhry R, Thakur CK. Determination of Cutoff of ELISA and Immunofluorescence Assay for Scrub Typhus. J Glob Infect Dis. 2016;8(3):97-9. Epub 2016/09/14. doi: 10.4103/0974-777x.188584. PubMed PMID: 27621559; PubMed Central PMCID: PMCPMC4997800.

42. Blacksell SD, Lim C, Tanganuchitcharnchai A, Jintaworn S, Kantipong P, Richards AL, et al. Optimal Cutoff and Accuracy of an IgM Enzyme-Linked Immunosorbent Assay for Diagnosis of Acute Scrub Typhus in Northern Thailand: an Alternative Reference Method to the IgM Immunofluorescence Assay. J Clin Microbiol. 2016;54(6):1472-8. Epub 2016/03/25. doi: 10.1128/jcm.02744-15. PubMed PMID: 27008880; PubMed Central PMCID: PMCPMC4879268.

43. Mørch K, Manoharan A, Chandy S, Chacko N, Alvarez-Uria G, Patil S, et al. Acute undifferentiated fever in India: a multicentre study of aetiology and diagnostic accuracy. BMC Infect Dis. 2017;17(1):665. Epub 2017/10/06. doi: 10.1186/s12879-017-2764-3. PubMed PMID: 28978319; PubMed Central PMCID: PMCPMC5628453.

44. Charoenphak S, Rattanawong P, Sungkanuparph S. Acute cholecystitis as an unusual presentation of scrub typhus: a report of two cases and review of the literature. Southeast Asian J Trop Med Public Health. 2017;48(1):143-9. Epub 2017/01/01. PubMed PMID: 29644830.

45. Tantibhedhyangkul W, Wongsawat E, Silpasakorn S, Waywa D, Saenyasiri N, Suesuay J, et al. Use of Multiplex Real-Time PCR To Diagnose Scrub Typhus. J Clin Microbiol. 2017;55(5):1377-87. Epub 2017/02/17. doi: 10.1128/jcm.02181-16. PubMed PMID: 28202789; PubMed Central PMCID: PMCPMC5405255.

46. Kocher C, Jiang J, Morrison AC, Castillo R, Leguia M, Loyola S, et al. Serologic Evidence of Scrub Typhus in the Peruvian Amazon. Emerg Infect Dis. 2017;23(8):1389-91. Epub 2017/07/21. doi: 10.3201/eid2308.170050. PubMed PMID: 28726619; PubMed Central PMCID: PMCPMC5547797.

47. Chao CC, Zhang Z, Belinskaya T, Thipmontree W, Tantibhedyangkul W, Silpasakorn S, et al. An ELISA assay using a combination of recombinant proteins from multiple strains of *Orientia* *tsutsugamushi*  offers an accurate diagnosis for scrub typhus. BMC Infect Dis. 2017;17(1):413. Epub 2017/06/12. doi: 10.1186/s12879-017-2512-8. PubMed PMID: 28601091; PubMed Central PMCID: PMCPMC5466769.

48. Rawat V, Singh RK, Kumar A, Saxena SR, Varshney U, Kumar M. Epidemiological, clinical and laboratory profile of scrub typhus cases detected by serology and RT-PCR in Kumaon, Uttarakhand: a hospital-based study. Trop Doct. 2018;48(2):103-6. Epub 2017/11/28. doi: 10.1177/0049475517743891. PubMed PMID: 29173047.

49. Premaratna R, Blanton LS, Samaraweera DN, de Silva GN, Chandrasena NT, Walker DH, et al. Genotypic characterization of *Orientia* *tsutsugamushi*  from patients in two geographical locations in Sri Lanka. BMC Infect Dis. 2017;17(1):67. Epub 2017/01/15. doi: 10.1186/s12879-016-2165-z. PubMed PMID: 28086810; PubMed Central PMCID: PMCPMC5237229.

50. Koralur M, Singh R, Varma M, Shenoy S, Acharya V, Kamath A, et al. Scrub typhus diagnosis on acute specimens using serological and molecular assays - a 3-year prospective study. Diagn Microbiol Infect Dis. 2018;91(2):112-7. Epub 2018/05/01. doi: 10.1016/j.diagmicrobio.2018.01.018. PubMed PMID: 29706479.

51. Ogawa M, Satoh M, Saijo M, Ando S. Evaluation of a broad-ranging and convenient enzyme-linked immunosorbent assay using the lysate of infected cells with five serotypes of *Orientia* *tsutsugamushi* , a causative agent of scrub typhus. BMC Microbiol. 2017;17(1):7. Epub 2017/01/07. doi: 10.1186/s12866-016-0910-5. PubMed PMID: 28056811; PubMed Central PMCID: PMCPMC5217197.

52. Faruque LI, Zaman RU, Gurley ES, Massung RF, Alamgir AS, Galloway RL, et al. Prevalence and clinical presentation of *Rickettsia*, *Coxiella*, *Leptospira*, *Bartonella* and chikungunya virus infections among hospital-based febrile patients from December 2008 to November 2009 in Bangladesh. BMC Infect Dis. 2017;17(1):141. Epub 2017/02/15. doi: 10.1186/s12879-017-2239-6. PubMed PMID: 28193163; PubMed Central PMCID: PMCPMC5307764.

53. Blacksell SD, Kingston HWF, Tanganuchitcharnchai A, Phanichkrivalkosil M, Hossain M, Hossain A, et al. Diagnostic Accuracy of the InBios Scrub Typhus Detect™ ELISA for the Detection of IgM Antibodies in Chittagong, Bangladesh. Trop Med Infect Dis. 2018;3(3). Epub 2018/10/03. doi: 10.3390/tropicalmed3030095. PubMed PMID: 30274491; PubMed Central PMCID: PMCPMC6160969.

54. Yang SL, Tsai KH, Chen HF, Luo JY, Shu PY. Evaluation of Enzyme-Linked Immunosorbent Assay Using Recombinant 56-kDa Type-Specific Antigens Derived from Multiple *Orientia* *tsutsugamushi*  Strains for Detection of Scrub Typhus Infection. Am J Trop Med Hyg. 2019;100(3):532-9. Epub 2018/12/12. doi: 10.4269/ajtmh.18-0391. PubMed PMID: 30526730; PubMed Central PMCID: PMCPMC6402918 scrub typhus and detection method thereof pending.

55. Kingston HW, Hossain M, Leopold S, Anantatat T, Tanganuchitcharnchai A, Sinha I, et al. Rickettsial Illnesses as Important Causes of Febrile Illness in Chittagong, Bangladesh. Emerg Infect Dis. 2018;24(4):638-45. Epub 2018/03/20. doi: 10.3201/eid2404.170190. PubMed PMID: 29553921; PubMed Central PMCID: PMCPMC5875266.

56. Tshokey T, Stenos J, Durrheim DN, Eastwood K, Nguyen C, Vincent G, et al. Rickettsial Infections and Q Fever Amongst Febrile Patients in Bhutan. Trop Med Infect Dis. 2018;3(1). Epub 2018/10/03. doi: 10.3390/tropicalmed3010012. PubMed PMID: 30274410; PubMed Central PMCID: PMCPMC6136613.

57. Sharma R, Mahajan SK, Singh B, Raina R, Kanga A. Predictors of Severity in Scrub Typhus. J Assoc Physicians India. 2019;67(4):35-8. Epub 2019/07/14. PubMed PMID: 31299836.

58. Kim HL, Park HR, Kim CM, Cha YJ, Yun NR, Kim DM. Indicators of severe prognosis of scrub typhus: prognostic factors of scrub typhus severity. BMC Infect Dis. 2019;19(1):283. Epub 2019/03/27. doi: 10.1186/s12879-019-3903-9. PubMed PMID: 30909868; PubMed Central PMCID: PMCPMC6434784.

59. Singla N, Mahajan V, Chander J, Guglani V. Clinicoepidemiological and Genotyping Correlation of Pediatric Scrub Typhus from Chandigarh, India. Indian Pediatrics. 2020;57:314-6.

60. Eisermann P, Rauch J, Reuter S, Eberwein L, Mehlhoop U, Allartz P, et al. Complex Cytokine Responses in Imported Scrub Typhus Cases, Germany, 2010-2018. Am J Trop Med Hyg. 2020;102(1):63-8. Epub 2019/11/27. doi: 10.4269/ajtmh.19-0498. PubMed PMID: 31769398; PubMed Central PMCID: PMCPMC6947778.

61. Wangrangsimakul T, Greer RC, Chanta C, Nedsuwan S, Blacksell SD, Day NPJ, et al. Clinical Characteristics and Outcome of Children Hospitalized With Scrub Typhus in an Area of Endemicity. J Pediatric Infect Dis Soc. 2020;9(2):202-9. Epub 2019/03/14. doi: 10.1093/jpids/piz014. PubMed PMID: 30864670; PubMed Central PMCID: PMCPMC7192406.

62. Phanichkrivalkosil M, Tanganuchitcharnchai A, Jintaworn S, Kantipong P, Laongnualpanich A, Chierakul W, et al. Determination of Optimal Diagnostic Cut-Offs for the Naval Medical Research Center Scrub Typhus IgM ELISA in Chiang Rai, Thailand. Am J Trop Med Hyg. 2019;100(5):1134-40. Epub 2019/03/13. doi: 10.4269/ajtmh.18-0675. PubMed PMID: 30860022; PubMed Central PMCID: PMCPMC6493932.

63. Thakur CK, Chaudhry R, Gupta N, Vinayaraj EV, Singh V, Das BK, et al. Scrub typhus in patients with acute febrile illness: a 5-year study from India. Qjm. 2020;113(6):404-10. Epub 2019/12/04. doi: 10.1093/qjmed/hcz308. PubMed PMID: 31790119.

64. Yen TY, Zhang Z, Chao CC, Ching WM, Shu PY, Tseng LF, et al. Serologic Evidence for *Orientia* Exposure in the Democratic Republic of Sao Tome and Principe. Vector Borne Zoonotic Dis. 2019;19(11):821-7. Epub 2019/08/14. doi: 10.1089/vbz.2018.2426. PubMed PMID: 31407963.

65. Elders PND, Dhawan S, Tanganuchitcharnchai A, Phommasone K, Chansamouth V, Day NPJ, et al. Diagnostic accuracy of an in-house Scrub Typhus Enzyme linked immunoassay for the detection of IgM and IgG antibodies in Laos. PLoS Negl Trop Dis. 2020;14(12):e0008858. Epub 2020/12/08. doi: 10.1371/journal.pntd.0008858. PubMed PMID: 33284807.

66. Pote K, Narang R, Deshmukh P. Diagnostic performance of serological tests to detect antibodies against acute scrub typhus infection in central India. Indian J Med Microbiol. 2018;36(1):108-12. Epub 2018/05/08. doi: 10.4103/ijmm.IJMM_17_405. PubMed PMID: 29735837.

67. Gautam R, Parajuli K, Tshokey T, Stenos J, Sherchand JB. Diagnostic evaluation of IgM ELISA and IgM Immunofluorescence assay for the diagnosis of Acute Scrub Typhus in central Nepal. BMC Infect Dis. 2020;20(1):138. Epub 2020/02/15. doi: 10.1186/s12879-020-4861-y. PubMed PMID: 32054525; PubMed Central PMCID: PMCPMC7020552.

68. Jain A, Jain P, Rebello SC, Todaria M, Kaur H, Gupta N, et al. Determination of a cut-off value for the serological diagnosis of scrub typhus by detecting anti-*Orientia* *tsutsugamushi*  immunoglobulin M. Indian J Med Res. 2023;157(6):519-23. Epub 2023/08/02. doi: 10.4103/ijmr.IJMR_4098_20. PubMed PMID: 37530306; PubMed Central PMCID: PMCPMC10466483.

69. Ogawa M, Ando S, Saijo M. Evaluation of Recombinant Type-Specific Antigens of *Orientia* *tsutsugamushi*  Expressed by a Baculovirus-Insect Cell System as Antigens for Indirect Immunofluorescence Assay in the Serological Diagnosis of Scrub Typhus. Jpn J Infect Dis. 2020;73(5):330-5. Epub 2020/05/01. doi: 10.7883/yoken.JJID.2019.334. PubMed PMID: 32350215.

70. de Vries SG, van Eekeren LE, van der Linden H, Visser BJ, Grobusch MP, Wagenaar JFP, et al. Searching and Finding the Hidden Treasure: A Retrospective Analysis of Rickettsial Disease Among Dutch International Travelers. Clin Infect Dis. 2021;72(7):1171-8. Epub 2020/01/31. doi: 10.1093/cid/ciaa091. PubMed PMID: 31998942; PubMed Central PMCID: PMCPMC8028097.

71. Varghese GM, Kannan K, Abhilash K, Nithyananth A, David T, Sathyendra S, et al. 1655. Performance of Molecular and Serologic Tests for the Diagnosis of Scrub Typhus. Open Forum Infectious Diseases. 2019;6(Supplement_2):S605-S. doi: 10.1093/ofid/ofz360.1519.

72. Kim CM, Kim DM, Yun NR. Evaluation of the Diagnostic Accuracy of Antibody Assays for Patients with Scrub Typhus. J Clin Microbiol. 2021;59(7):e0294220. Epub 2021/04/23. doi: 10.1128/jcm.02942-20. PubMed PMID: 33883180; PubMed Central PMCID: PMCPMC8218765.

73. Roy S, Yadav S, Garg S, Deshmukh PR, Narang R. Evaluation of nested PCR and loop mediated isothermal amplification assay (LAMP) targeting 47 ​kDa gene of *Orientia* *tsutsugamushi*  for diagnosis of scrub typhus. Indian J Med Microbiol. 2021;39(4):475-8. Epub 2021/07/04. doi: 10.1016/j.ijmmb.2021.06.011. PubMed PMID: 34215476.

74. Mansoor T, Fomda BA, Koul AN, Bhat MA, Abdullah N, Bhattacharya S, et al. Rickettsial Infections among the Undifferentiated Febrile Patients Attending a Tertiary Care Teaching Hospital of Northern India: A Longitudinal Study. Infect Chemother. 2021;53(1):96-106. Epub 2021/08/20. doi: 10.3947/ic.2020.0147. PubMed PMID: 34409783; PubMed Central PMCID: PMCPMC8032907.

75. Um J, Nam Y, Lim JN, Kim M, An Y, Hwang SH, et al. Seroprevalence of scrub typhus, murine typhus and spotted fever groups in North Korean refugees. Int J Infect Dis. 2021;106:23-8. Epub 2021/03/07. doi: 10.1016/j.ijid.2021.02.111. PubMed PMID: 33676004.

76. Chaudhari SP, Kalorey DR, Awandkar SP, Kurkure NV, Narang R, Kashyap RS, et al. Journey towards National Institute of One Health in India. Indian J Med Res. 2021;153(3):320-6. Epub 2021/04/29. doi: 10.4103/ijmr.IJMR_636_21. PubMed PMID: 33906994; PubMed Central PMCID: PMCPMC8204833.

77. Kim CM, Kim DM, Yun NR. Follow-up investigation of antibody titers and diagnostic antibody cutoff values in patients with scrub typhus in South Korea. BMC Infect Dis. 2021;21(1):69. Epub 2021/01/15. doi: 10.1186/s12879-020-05735-8. PubMed PMID: 33441087; PubMed Central PMCID: PMCPMC7807423.

78. Devamani CS, Prakash JAJ, Alexander N, Stenos J, Schmidt WP. The incidence of *Orientia* *tsutsugamushi*  infection in rural South India. Epidemiol Infect. 2022;150:e132. Epub 2022/06/30. doi: 10.1017/s0950268822001170. PubMed PMID: 35765168; PubMed Central PMCID: PMCPMC9306010.

79. Patil S, Patil A, Chaudhari S, Shinde S, Kolte S, Khan W, et al. Edifications on Indirect IgM ELISA and Immunofluorescence assay (IFA) of scrub typhus in humans and erudition of *Orientia* *tsutsugamushi*  in the vector of cohabiting rodents. J Vector Borne Dis. 2023;60(3):244-51. Epub 2023/10/16. doi: 10.4103/0972-9062.355964. PubMed PMID: 37843234.

80. Chaisiri K, Tanganuchitcharnchai A, Kritiyakan A, Thinphovong C, Tanita M, Morand S, et al. Risk factors analysis for neglected human rickettsioses in rural communities in Nan province, Thailand: A community-based observational study along a landscape gradient. PLoS Negl Trop Dis. 2022;16(3):e0010256. Epub 2022/03/24. doi: 10.1371/journal.pntd.0010256. PubMed PMID: 35320277; PubMed Central PMCID: PMCPMC8979453.

81. Narang R, Deshmukh P, Jain J, Jain M, Raut A, Deotale V, et al. Scrub typhus in urban areas of Wardha district in central India. Indian J Med Res. 2022;156(3):435-41. Epub 2023/01/03. doi: 10.4103/ijmr.IJMR_707_19. PubMed PMID: 36588363; PubMed Central PMCID: PMCPMC10101365.

82. Tasak N, Apidechkul T, Law ACK, Abdad MY, Srichan P, Perrone C, et al. Prevalence of and factors associated with scrub typhus exposure among the hill tribe population living in high incidence areas in Thailand: a cross-sectional study. BMC Public Health. 2023;23(1):2394. doi: 10.1186/s12889-023-17313-z.

83. Faccini-Martínez Á A, Silva-Ramos CR, Blanton LS, Arroyave E, Martínez-Diaz HC, Betancourt-Ruiz P, et al. Serologic Evidence of *Orientia* Infection among Rural Population, Cauca Department, Colombia. Emerg Infect Dis. 2023;29(2):456-9. Epub 2023/01/25. doi: 10.3201/eid2902.221458. PubMed PMID: 36692499; PubMed Central PMCID: PMCPMC9881757.

84. Narayanappa D, Geetha R, Rajani HS. Diagnostic Accuracy of Rapid Antibody Detection Test for Scrub Typhus. Indian Pediatr. 2023;60(7):546-8. Epub 2023/07/10. PubMed PMID: 37424119.
